# PB.44. Do qualitative patterns of stiffness help differentiate benign from malignant breast masses of similar stiffness during shear wave elastography?

**DOI:** 10.1186/bcr3740

**Published:** 2014-11-03

**Authors:** M Elseedawy, P Whelehan, S Vinnicombe, D McLean, K Thomson, A Evans

**Affiliations:** 1 University of Dundee, UK; 2 Dundee Cancer Centre, Dundee, UK; 3 Ninewells Hospital, Dundee, UK

## Introduction

Previous studies assessing qualitative patterns of stiffness in breast lesions have not matched benign and malignant lesions for quantitative stiffness. The purpose of this study is to compare the qualitative patterns of stiffness of benign and malignant lesions matched for quantitative stiffness.

## Methods

A total of 158 consecutive histologically confirmed benign lesions with a mean stiffness >30 kPa were identified from a prospective database. Forty-nine cancers with the same distribution of stiffness as the benign lesions were identified for comparison. The following features were assessed by two observers; BIRADS score, whether there was stiffness in the lesion, outside the lesion, Tozaki classification, homogeneity of stiffness, the presence of a ring sign, stiffness adjacent to the skin or muscle, and the assessed likelihood of the stiffness being artefactual. Statistical analysis was carried out using the chi-square test.

## Results

Benign and malignant lesions both had a mean stiffness of 73 kPa. The following features show a significant association with malignancy; BIRADS classification 4 or 5 (45 (91%) vs. 99 (63%), *P *< 0.0001), Tozaki classification 3 and 4 (43 (88%) vs. 85 (54%), *P *< 0.001), and presence of a ring sign (40 (82%) vs. 78 (49%), *P *< 0.001). The following features were more common in benign lesions; stiffness in the lesion (103 (65%) vs. 17 (35%), *P *= 0.0002), stiffness adjacent to skin (99 (63%) vs. 19 (39%), *P *= 0.003) and stiffness thought to be probably artefact (74 (47%) vs. 5 (10%), *P *< 0.0001).

## Conclusion

Qualitative features show significant differences in frequency when comparing benign and malignant masses. Qualitative features could be used to help classify breast lesions during SWE.

